# Artificial intelligence in medicine and the disclosure of risks

**DOI:** 10.1007/s00146-020-01085-w

**Published:** 2020-10-22

**Authors:** Maximilian Kiener

**Affiliations:** The Queen’s College, Faculty of Philosophy, The University of Oxford, High Street, Oxford, OX1AW UK

**Keywords:** Artificial intelligence, Medical disclosure, Risks, Informed consent, COVID-19

## Abstract

This paper focuses on the use of ‘black box’ AI in medicine and asks whether the physician needs to disclose to patients that even the best AI comes with the risks of cyberattacks, systematic bias, and a particular type of mismatch between AI’s implicit assumptions and an individual patient’s background situation. *Pace* current clinical practice, I argue that, under certain circumstances, these risks do need to be disclosed. Otherwise, the physician either vitiates a patient’s informed consent or violates a more general obligation to warn him about potentially harmful consequences. To support this view, I argue, first, that the already widely accepted conditions in the evaluation of risks, i.e. the ‘nature’ and ‘likelihood’ of risks, speak in favour of disclosure and, second, that principled objections against the disclosure of these risks do not withstand scrutiny. Moreover, I also explain that these risks are exacerbated by pandemics like the COVID-19 crisis, which further emphasises their significance.

## Introduction

Artificial intelligence (AI) increasingly executes tasks that previously only humans could do, such as driving a car or even performing complicated medical procedures. What is more, AI also outperforms humans in these tasks. On average, AI is the better driver and in some domains of medical diagnosis (Bathaee 2018), drug development (Arshadi et al. 2020), and even the execution of treatment and surgery (Ficuciello et al. 2019; Ho 2020), AI already is—or soon promises to be—better than trained medical professionals.

Unfortunately, the best AI also tends to be the least transparent, often resulting in a ‘black box’ (Carabantes 2019). We can see which data go into the AI system and also which come out. We may even understand how such AI systems work in general terms, i.e. usually through so-called deep neural networks. Yet, we often cannot understand why, on a certain occasion, the AI system made a particular decision, arrived at a particular diagnosis, or performed a particular move in an operation (Bathaee 2018; Carabantes 2019; Coeckelbergh 2020). This is because of the sheer complexity of these systems, which may base a single output on as many as 23 million parameters, e.g. ‘Inception v3’ developed by Google (Wang et al. 2019), and the fact that AI systems constantly change their own algorithms without human supervision (Bathaee 2018; Price 2017). Although there is growing research on so-called “eXplainable AI” (“XAI”) (Samek 2019), many aspects of AI are still *un*-explainable and, given the increasing sophistication of AI, they are likely to remain so in the future too.

In this paper, I will focus on such non-transparent or, as I will also call it, *black box AI* in medicine. More precisely, I will ask whether the physician needs to disclose to patients that even the best AI comes with the risks of cyberattacks, systematic bias, and a particular type of mismatch. *Pace* current clinical practice, I will argue that, under certain circumstances, these risks do need to be disclosed to a patient. Otherwise, the physician either vitiates the patient’s informed consent or violates a more general obligation to warn him about potentially harmful consequences.

I will proceed as follows. I will first explain the significance of medical disclosure and endorse two widely accepted conditions in the evaluation of risks: the ‘nature’ and ‘likelihood’ of risks (2). I will then discuss each of the three aforementioned risks in turn. For each risk, I will argue that the conditions of ‘nature’ and ‘likelihood’ speak in favour of disclosure and that a more principled objection against disclosing these risks does not withstand scrutiny. I will also point out that these risks are exacerbated in a pandemic like the COVID-19 crisis (3). Finally, I will summarise my results (4).

## Medical disclosure and the assessment of risks

People have a right to self-determination, which includes a right to decide how their own body will be treated. This means that competent individuals, i.e. individuals possessing the required mental capacities, have the right to decide which available medical procedures to undergo. Consent is a tool for exercising such a right and one by which individuals grant their physicians or clinical investigators permission to perform a medical procedure, which it would otherwise be impermissible to perform. But in order for a competent person’s consent to be valid, it has to be *informed*, which means that the individual needs to know the relevant facts about the medical procedure.

Thus, the first and most important reason why medical disclosure is significant is to ensure that a patient’s consent is sufficiently informed. Only if a physician discloses relevant details about a medical procedure or condition to his patients can the latter make informed decisions about their health care and thereby give valid consent; and since valid consent is key to modern clinical practice, medical disclosure of relevant information is highly significant too.

Yet, the need for medical disclosure is not restricted to informed consent. As Walker pointed out, only interferences with a patient’s body normally require informed consent, but not other actions in the medical context. For example, there is no requirement of consent to the prescription of medication (Walker 2017), but in such a context, physicians may still be under an obligation to disclose relevant risks and provide information. Here, the obligation to disclose relevant information is part of a wider obligation not to warn a patient about potentially harmful consequences.

Thus, when discussing medical disclosure in the context of AI in this paper, I will focus not only on informed consent but also on a more general obligation to disclose relevant information to patients. Yet, as the most interesting AI applications in medicine link to medical procedures which require informed consent, my main focus will still be on disclosure in the context of consent.

With this clarification of the significance of medical disclosure at hand, I will now examine it in greater detail. Medical disclosure normally requires physicians to inform their patients about the risks, benefits, and potential alternatives that a medical procedure or course of action has. In this paper, however, I will particularly focus on risks; and indeed, in the academic literature, scholars have stressed that the disclosure of risks is particularly important and necessary for patients to make a rational and balanced decision.^1^ Which particular risks require disclosure has been subject to extensive legal and ethical debate (McLean 2010, 47ff). But it is generally agreed that a requirement of disclosure can be determined on the basis of two criteria: the ‘nature’ and the ‘likelihood’ of a risk (Beauchamp and Childress 2013, 125ff; Berg and Applebaum 2001, 46–58; Herring 2016, 174–178; Maclean 2009, 135–136, 163–177; McLean 2010, 42–47, 73–81).

The nature of the risk is what the risk is a risk *of* or, put another way, how bad the potential consequences of a medical procedure are. For instance, the risk of paralysis is worse and therefore more significant than the risk of a bruise. However, to assess the nature of the risk, we cannot merely look at the objective physical or psychological harm but must always also take into account the relevance of certain types of harm for individuals.^2^ For instance, we need to take into account that a concert pianist is likely to be especially concerned about a slight paralysis of his hands.

On the other hand, the likelihood of a risk is the probability of the harm occurring. Risks become more significant the more likely they are to materialise.^3^ However, following the influential legal case of *Montgomery v Lanarkshire Health Board (2015)*, legal scholars and philosophers alike have claimed that even very low risks can require disclosure if a reasonable person would consider the risk material to his decision, e.g. a risk of paralysis should be disclosed even if it is considerably below 1% (Lee and Lai 2020). Moreover, just as in the assessment of the nature of a risk, the assessment of the likelihood also requires taking into account individual perspectives. In other words, we need to take into account not only what the average person would consider significant but also what an individual, potentially risk-averse patient may consider significant.

Thus, risks require disclosure when their nature and likelihood are beyond a certain threshold, as measured from the perspective of the reasonable *and* individual consenter. In its general form, this view is widely shared and, in what follows, I will rely on it to support my claims. More precisely, using the criteria of ‘nature’ and ‘likelihood’, I will describe three characteristic risks of black box AI, viz. cyber-attacks, systematic bias, and a particular type of mismatch; I will also explain why these risks are exacerbated by pandemics like the current COVID-19 crisis and argue that such risks need to be disclosed to patients under certain circumstances.

## Three risks of black box AI

### Cyber-attacks

AI systems are computer systems and, therefore, subject to the risk of cyber-attacks. In fact, academic research is continuously uncovering new ways in which current state-of-the-art AI can be attacked, highlighting the growing importance of cybersecurity in medicine (Elsayed et al. 2018; Ilyas et al. 2018; Nguyen et al. 2015; Yao et al. 2019). Attacks on AI systems can undermine diagnostic accuracy, administer lethal drug doses, or sabotage critical moves in an operation (Finlayson et al. 2019; Hutson 2018; Kim et al. 2019; Sun et al. 2018). In this section, I will focus on one particular type of cyber-attack on AI, i.e. ‘input attacks.’ Such attacks are especially relevant because they illustrate that, even though cyber-risks are not restricted to AI systems and are a much wider problem, AI systems display a distinctively new cyber-vulnerability and this, therefore, necessitates a readjustment of medical disclosure too.

‘Input attacks’ manipulate the data entering the AI system so that it will deliver a wrong result (Comiter 2019). For instance, an attacker could change the pixel value of an MRI scan so that the AI system will categorise tissue as falsely malignant with a confidence rate of over 99% when it would correctly categorise it as benign with the same confidence rate in the absence of the attack (Finlayson et al. 2019). As a result, a patient may receive a false diagnosis, potentially leading to unnecessary chemotherapy or surgery, or even suffer great harm when otherwise necessary surgery is misdirected through manipulated medical images, e.g. a patient could be blinded in eye surgery or paralysed in spinal surgery.

These input attacks are considerably different from traditional cyber-attacks. Most importantly, they no longer need to interfere with or hack the AI system itself. Unlike other cyber-attacks, input attacks no longer compromise their target system. The AI system itself, its algorithm, and how it works can be left completely untouched. *Input attacks* only need to manipulate the *input data*. Hence, unlike traditional cyberattacks, “[input] attacks are not bugs in the [AI system’s] code that can be fixed—they are inherent in the heart of the AI algorithms” (Comiter 2019, 4).

In addition, input attacks are extremely hard to detect. They only require very subtle changes to a medical image, completely undetectable for the human eye. Attackers only need to scatter some digital dust over the image in the right places. Moreover, since input attacks do not interfere with the AI system itself, there will not be any traces in the AI system either. The only way to detect an attack is to detect the intrusion in another computer system where the medical images have been stored (Comiter 2019, 15; Finlayson et al. 2019, 1287). But even here, one might be unable to tell whether, in addition to the intrusion into the data base and the potential theft of medical records, attackers made any changes to medical images at all, why they might have done so, and what consequences such changes could have.

The nature of this risk of an input attack could indeed be significant. When subject to a cyber-attack, a patient might not only be severely *harmed* but also *wronged;* and being wronged, i.e. having a right violated, holds a special significance compared to being merely harmed, i.e. having one’s interests encroached upon. Compare the situation where someone accidentally steps on your toes (i.e. merely harms you) with the situation where someone deliberately hurts you (i.e. also wrongs you). We attach greater significance to being wronged and harmed as opposed to being merely harmed because the former not only encroaches upon some of our interests, but also calls into question our status as someone worthy of respect (Darwall 2006). Thus, the nature of the risks of cyber-attacks can speak in favour of disclosure, at least when a patient’s health critically relies on AI systems, e.g. in the case of surgery.

Concerning the likelihood of the risk of a cyberattack, we know that “[c]yberattacks on medical devices and hospital networks are a real and growing threat” (Wellington 2014, 139) (Argaw et al. 2019; Lallie et al. 2020; Wirth 2020). Recent attacks on hospitals in the UK and the Czech Republic confirm this (Clarke and Youngstein 2017; O'Dowd 2017). Moreover, we also know that cyber-attacks become more frequent in times of pandemics (Argaw et al. 2019; Lallie et al. 2020; Wirth 2020). During the current COVID-19 crisis, we have seen numerous attacks in France, Spain, the United States, and other countries. Some forms of traditional cyber-attacks have even increased by 600% since the start of the COVID-19 crisis (Lallie et al. 2020). Therefore, insofar as empirical evidence for the likelihood of cyber-attacks is concerned, we should take the risk of cyber-attacks very seriously.

Thus, on the basis of the nature and likelihood of the risk of an input attack on AI, disclosure could indeed be required, at least in certain cases where the nature and likelihood of the risk become significant. Failing to disclose this risk could, therefore, vitiate informed consent or violate the physician’s obligation to warn patients about potentially harmful consequences of certain procedures.

However, there may be a principled objection to this conclusion. A number of US legal cases have argued that risks require disclosure only if they are ‘inherent’ in a medical procedure (see *Gilmartin v Weinreb, Calabrese v Trenton State College, Battenfeld v Gregory, Gracia v Meiselman, Barclay v Campbell, and Jones v Papp*). A risk is ‘inherent’, the judges in *Jones v Papp* explained, if and only if the “risk is one which exists in and is inseparable from the procedure itself” (*Jones v Papp*). Conversely, a physician does not have a duty to disclose the risks and “dangers in the procedure if not done properly” (*Mallett v Pirkey*).^4^ Even though these cases focused on the risk of negligent action by physicians and argued that such risks are not ‘inherent’ so as to require disclosure, the conclusions from these cases seem to apply *a fortiori* to the present context too. If risks require disclosure only when they are ‘inherent’ in a procedure, then risks arising from some cyber-interference committed by third-parties do not need to be disclosed either. They are simply not a risk *of the* procedure. They are *criminal* risks.

This objection raises an important point. There is indeed a connection between the requirement of disclosure and the expertise of a physician *qua* medical professional. Physicians need to disclose inherent medical risks because their medical expertise puts them, unlike laypeople, in a privileged position to know about these risks in the first place. However, physicians cannot be expected to make predictions about the likelihood of certain people attacking their patients through cyber-attacks. After all, physicians are not criminologists. Thus, they are not in a position, let alone obliged, to disclose *those* risks.

On the other hand, this objection underestimates the new quality of input attacks. As explained earlier, input attacks leave the AI system completely uncompromised. The AI system would still work normally, not be subject to any bug or interference, and the physician performing or supervising the procedure would not fall short of expected professional conduct. Therefore, *none* of such AI-based procedures can avoid the vulnerability to input attacks. But if this is so, then the risk of input attacks does become ‘inherent’ to certain medical procedures, as defined earlier: it is a “risk (…) which exists in and *is inseparable from* the [AI-based] procedure itself” (Jones v. Papp; emphasis added). Therefore, Comiter was right when he claimed that the risk of input attacks is “inherent in the heart of the AI algorithms” (Comiter 2019, 4) rather than something that only affects a few less secure algorithms.

Thus, even though the objection at hand makes a valid point about other criminal risks that are *external* to medical procedures, it fails to appreciate the fact that the risk of input attacks became inseparable from certain AI-based procedures and therefore an ‘inherent’ risk that requires disclosure. After all, physicians are required to know about risks that are inseparable from a particular medical procedure and need to disclose them to their patients.

Before I conclude, however, let me add two further arguments to support my view in favour of disclosing the risk of input attacks. Firstly, consider the widely endorsed view developed in the legal case *Montgomery v Lanarkshire Health Board (2015)*, i.e. the view that risks require disclosure when a reasonable person would want to know about them. And to clarify, I will only focus on the claim of my hypothetical objector that cyber-risks do not need to be disclosed due to their *criminal* dimension. So, would a reasonable person really not want to be informed about a risk of an input attack, which is specific to and ‘inherent’ in a medical procedure simply because it is not a *purely medical* risk but also one that has a *criminal* dimension? I doubt it. Given the special significance that wronging has for us, as outlined earlier, the mere fact that we are dealing with a criminal aspect does not make it less significant for a reasonable person. Thus, the test of the reasonable person supports my conclusion in favour of disclosing the risk of an input attack.

Second, let me reframe the question from ‘which *risks* require disclosure’ to ‘which *alternative* forms of treatment require disclosure’. Suppose there are two treatments available: one operates with an AI system that is subject to the risk of an input attack but also provides a further medical benefit, whereas the other operates without such AI, thereby sidesteps the risk of an input attack, but then also fails to provide the additional medical benefit. Furthermore, and to make other things equal, suppose that all other features of the two treatments are identical. It seems that the physician has to inform the patient about both alternatives: if there is an additional available medical benefit, physicians need to inform their patients about it and allow them to decide whether to take advantage of it. However, adequately informing the patient requires that physicians not only point to the medical benefit but also highlight the potential risk that comes with it (assuming that it is significant in terms of its nature and likelihood). Otherwise, the patient’s decision in favour of the medical benefit would be one-sided and flawed. Thus, by switching the perspective to the disclosure of *alternatives*, I also reach the conclusion that the risk of input attacks requires disclosure.

Hence, the view that only ‘inherent’ risks require disclosure does not create a principled objection to disclosing the risk of input attacks. The risk of input attacks is in fact ‘inherent’ in certain AI-based medical procedures in the relevant sense and the fact that it is also a criminal risk does not undermine the need to disclose it.

### Systematic Bias

Medical AI is trained with a large amount of data, but the size of such data does not prevent the AI from being biased and posing the risk of false results.^5^ To begin with, AI may be biased because the training set was not sufficiently diverse. If so, the AI system only works accurately on those people who match the input data but performs poorly on those who do not. As Parikh and colleagues explain:

“For example, among women with breast cancer, black women had a lower likelihood of being tested for high-risk germline mutations compared with white women, despite carrying a similar risk of such mutations. Thus, an AI algorithm that depends on genetic test results is more likely to mischaracterize the risk of breast cancer for black patients than white patients” (Parikh et al. 2019, 2377) See also: (Carter et al. 2020; Challen et al. 2019; Char et al. 2018; Obermeyer et al. 2019; Popejoy and Fullerton 2016; Reddy et al. 2020).

Thus, AI may be biased against certain minorities, especially those who are already disadvantaged in society, and provides worse healthcare for them. This can be seen as a form of unintended discrimination and injustice, i.e. another instance of harming *and* wronging (Obermeyer et al. 2019; Popejoy and Fullerton 2016).

In addition, medical AI may be biased because the individual decisions of human physicians, which the AI is often trained with, were themselves flawed and marked by prejudice. Here, we face a ‘bias in, bias out’ scenario: if AI is trained with biased input, it will produce biased output. As Parikh and colleagues illustrate:

“Clinicians may incorrectly discount the diagnosis of myocardial infarction in older women because these patients are more likely to present with atypical symptoms. An AI algorithm that learns from historical electronic health record (EHR) data and existing practice patterns may not recommend testing for cardiac ischemia for an older woman, delaying potentially life-saving treatment.” (Parikh et al. 2019, 2377).

Hence, in these situations, the problem is not that the training data, taken as whole, were insufficiently diverse but that the training data, taken separately, were flawed. AI cannot but conform to the prejudiced judgments it was trained on.

This risk of harm through bias is particularly pertinent to black box AI because its opacity makes it extremely difficult, if not impossible, to detect the effect of bias on individual outputs. We simply cannot look inside its decision-making processes on particular occasions to check what exactly determined a certain output. Moreover, the risk of harm through bias is also further exacerbated in pandemics like the COVID-19 crisis. Such a pandemic is a global crisis spanning numerous countries with a maximally diverse group of patients. Yet, despite the best efforts of computer scientists, the training data of AI systems are likely to be derived from a small and much more homogenous subset of people. Moreover, the rapid rise of COVID-19 and the time pressure involved in finding a solution may further increase the difficulty of securing a sufficiently diverse data set. Thus, the risk of bias from insufficient diversity is particularly high. In addition, individually prejudiced training data are also more likely to cause harm because there is a greater number of ill people in pandemics and therefore a greater likelihood that the group of patients will also include those who are most vulnerable to individually flawed training data. Thus, does the risk of harm through bias require disclosure in a situation like the current COVID-19 crisis?

The nature and likelihood of such risks may speak in favour of disclosure. Concerning the ‘nature’ of these risks, there is a possibility of great harm, e.g. in cases of false diagnoses and subsequent harmful procedures, or more direct AI interferences with a patient’s body. Moreover, the fact that one could receive worse healthcare simply because of certain features, such as race or sex, make this a potential case of unjust discrimination, i.e. a potential case of wronging (even if unintended), and thereby gives special significance to such harm and discrimination. On the other hand, concerning the ‘likelihood’ of these risks, we can be certain that, in some form or another, AI systems *will* be biased and the extent of such bias may equate to the extent of bias in current *human* medical decision-making. Thus, it seems that the two parameters suggest that the risks of harm due to AI bias can be significant and therefore require disclosure.

But there is a yet more principled objection: human physicians have also been trained on an insufficiently diverse data set. Moreover, their individual judgments are also flawed and marked by prejudice. However, in current clinical practice, they are not required to disclose their biases or the risks associated with them and this practice seems justified. Hence, one could employ an argument from analogy at this point and claim that the risk of harm through AI biases does not need to be disclosed either.

This objection misses two important aspects. First, psychological research has shown that people have an ‘automation bias’, i.e. people display an unjustified reliance on machines over human decisions and are likely to think that AI is free from the frailties of human choice (Goddard et al. 2012). As a result, not disclosing the risks of AI bias is likely to induce false beliefs in patients, whereas not disclosing the risk of bias in human physicians is not. After all, people know very well that human decision-makers, including physicians, are subject to bias and decision-making flaws. Therefore, to avoid inducing false beliefs, we may need to disclose the risk of bias in AI even though we do not need to disclose the risk of bias in physicians.

Second, when AI causes harm through bias, physicians are not responsible for such harm in the same way as they would be had their own biases caused it.^6^ This is because AI-caused harm is no longer attributable to any of the physician’s individual decisions. Rather, it has become a risk ‘inherent’ in the AI-based procedure, to use the formulation from the previous section. And this is an important shift. Patients can no longer hold physicians accountable for such harm *qua* something that *the physician* has done wrong. They must accept it as an inherent risk of AI-assisted medicine. But as a result, the content of valid consent changes. Insofar as people consent validly, patients assume responsibility for the risk of AI bias as something inherent in the medical procedure, i.e. a risk which was previously part of the physician’s responsibility. They thereby forego a later claim to complain when the risk materialises, at least in those cases where the physicians did not fall short of any professional norm. Hence, on the grounds of this change in what valid consent entails, the risk of bias in AI requires disclosure even though the risk of bias in physicians does not.

Thus, I conclude that the principled objection based on a parallel between physician bias and AI bias cannot defeat a requirement to disclose AI bias. In some cases, the nature and likelihood of the risks of bias still speak in favour of disclosing the risk of systematic bias.

### Mismatch

Big data have entered medicine. As Rajkomar and colleagues state: “routinely collected patient healthcare data are now approaching the genomic scale in volume and complexity” (Rajkomar et al. 2018, 1). What is more, big data may revolutionise medicine. The systematic use of such data, especially electronic health records (EHR), could considerably improve healthcare by personalising treatment to individual patients as well as significantly reduce costs by allocating medical resources more accurately (Bates et al. 2014; Jameson and Longo 2015; Krumholz 2014; Parikh et al. 2016, 2017). Yet, so far, the promised benefits have been closer to aspiration than reality: medical professionals still lack the tools and time to integrate big data into clinical practice (Choi et al. 2016).

New AI systems may change this situation. Currently, one of the most widely discussed uses of AI is risk prediction: using big data, AI systems become increasingly able to predict, for instance, how likely it is that individual patients will die in the near future (Aczon et al. 2017), how likely it is that they will develop a severe form of specific diseases like pneumonia (Caruana et al. 2015), or how likely it is that they will suffer certain complications, such as acute kidney failure (Tomašev et al. 2019). On the basis of such predictions, physicians are in a position to take the necessary steps to avoid harm for the individual patient, make a real difference to these people’s health, and also reduce healthcare costs.

However, even the best performing AI systems come with a serious challenge, viz. what I will call the risk of a *mismatch*. Since these AI systems are still insufficiently sensitive to causation as opposed to correlation, they may sometimes recommend courses of action that *do not match* the background situation of the individual patient, potentially leading to great harm. Consider the following example: Caruana and colleagues looked at how AI systems predicted the likelihood of death in patients with pneumonia. Here the AI was supposed to help clinicians to make a decision as to whether a certain person should be admitted to hospital or treated as an outpatient. Surprisingly, the AI assumed that asthmatic patients have a lower risk of developing severe pneumonia than those patients have who do not have asthma, i.e. an assumption which clearly conflicts with confirmed medical knowledge and, if relied on, could have potentially fatal consequences.

But interestingly, the AI system still detected a significant statistical correlation: asthmatic patients *normally* receive a certain treatment anyway and such treatment lowers the risk of developing severe pneumonia. Thus, given standard clinical practice, asthmatic patients *do* have a lower risk compared to non-asthmatic patients. As London comments:

“If the goal is to identify patients most at risk of dying *given standard practice*, then systems that rank asthmatics at lower risk are not biased. Rather, the system is actuarially correct—patients with asthma *who receive aggressive medical intervention* have a lower probability of death than some nonasthmatic patients who likely receive less aggressive medical care” (London 2019, 19).

Yet, having asthma is not the *cause* of the lower risk. It *merely correlates* with it, at least as long as standard clinical practice is in place. But unfortunately, and as already mentioned, even the best AI system cannot distinguish well between what *merely correlates* with the lower risk (i.e. having asthma) and what *causes* that lower risk (i.e. the standard treatment for asthmatics). As a result, the AI system may only work well for those asthmatics who receive standard care, but not for those who do not receive standard care. Hence, we face the risk of a *mismatch* between the circumstances of an individual patient and the implicit assumptions that AI makes in its calculation, e.g. that asthmatics receive standard care.

Caruana and colleagues detected this risk in ‘white box’ AI, where one could actually look into AI’s decision-making processes in detail. However, they also showed that this risk arises in black box AI too, where it is “virtually impossible to see inside” (Adadi and Berrada 2018, 52,144) the decision-making processes and, thus, where we will be unable to detect instances of a dangerous mismatch. Hence, the risk at hand is particularly serious in the case of black box AI and one that medical professionals and computer scientists alike may be unable to prevent.

The nature of this risk can be severe. As the pneumonia case showed (and many other examples could easily be added), it can be a matter of life or death. Data are currently sparse for the likelihood of the risk of a mismatch, yet we still know some things. The AI systems used in this context are far from being infallible (Choi et al. 2016) and part of their error rate includes cases in which harm occurred due to AI giving a treatment recommendation that did not match an individual patient’s background situation. Moreover, we can also infer that this risk must be exacerbated in a pandemic like the current COVID-19 crisis because the normal pre-pandemic conditions in health care, which the AI system will be trained on, are very likely to differ from the exceptional circumstances that arise during a pandemic (Turnham et al. 2020). Hence, when AI is applied to cases of pneumonia, as it actually is in the current COVID-19 pandemic (Cohen et al. 2020), the risk of a mismatch is indeed significant and, on these grounds, requires disclosure.

However, there is again a principled objection against disclosing this risk. One may argue that it is categorially different from the ones discussed so far. The risk of a mismatch is not an ‘inherent’ risk in a medical procedure, but rather—as I will put it—a *meta-risk*: it is a risk that the risk assessment is false. But such meta-risks do not normally require disclosure. For instance, physicians do not need to disclose the risk that the types of clinical research on which they rely when informing their patients could have been scientifically flawed and, therefore, have led to an erroneous assessment. But if physicians do not need to disclose such a risk, then they do not need to disclose the risk of a mismatch in the context of AI either.

This objection makes an important point and I agree that, normally, meta-risks do not require disclosure. Yet, I think that the situation is different in the context of AI. By way of explanation, big data and AI render risk assessments increasingly *personalised,* i.e. specifically tailored to a patient’s personal characteristics. This should be disclosed to patients because it will enable them to appreciate the improved overall accuracy of such an assessment, compared to a *general* risk assessment, and also mitigate what psychologists call an ‘optimism bias’, i.e. people’s tendency to think that they are less likely to be affected by negative events than the average person (Shepperd et al. 2002). In medicine, optimism bias makes people more likely to disregard *general* risks without good reason than they would *personalised* risks. Thus, telling patients that their risk assessment is personalised can reduce optimism bias as a potentially distorting factor in their decision-making. However, the adequate disclosure of the personalised character of the risk assessment must not only stress its advantages but also its potential flaws. Physicians need to tell their patients that even the best personalised risk assessment is not infallible and explain that one of the key reasons for error and harmful results is the risk of a mismatch, as described earlier. Hence, the first reason why physicians ought to disclose the risk of a mismatch (despite its status as a meta-risk) is that a balanced disclosure of the risk assessment’s personalised nature requires it.

In addition, there is also a second reason why the risk of a mismatch should be disclosed. As already mentioned, the risk of a mismatch is higher in a pandemic because clinical practice in a pandemic will likely deviate from the standard pre-pandemic clinical practice which AI systems assume to be in place. Thus, in pandemics, physicians must not present AI’s predictions to patients without qualification. Rather, they should highlight the uncertainty about the exact risk rates and explain that such uncertainty is due to an increased risk of a mismatch. If physicians fail to do this, they impede their patients’ understanding of the situation and are likely to present the patients with false information. Hence, the second reason why the risk of a mismatch should be disclosed is that adequate risk disclosure in extraordinary circumstances, as they arise in a pandemic especially, requires it.

However, before I conclude, I would like to stress that the risk of a mismatch need not always be a meta-risk and, at least in the future, could become an inherent risk in some medical procedures too. Suppose electronic health records are not only used when deciding on the length of hospitalisation but also when determining the exact moves in surgery or the exact doses of drug treatment. For instance, it could be the case that an AI system recommends a higher dose of a drug for an asthmatic patient because it assumes that the adverse effects of such a higher dose are neutralised by another drug which asthmatic patients *normally* receive. And as before, this assumption may work for most asthmatics but could lead to severe consequences for others. Alternatively, consider AI systems that determine the precise course of an operation on the basis of a personalised risk assessment. Again, the AI may rely on a certain correlation that fails to match that particular patient’s situation and thereby lead to adverse consequences too. Moreover, with the growing use of AI in medicine, such systems will also increasingly make decisions without human supervision and thereby lead more directly to harm in cases of a mismatch. Therefore, the risk of a mismatch can indeed become inherent in certain medical procedures in the future and, if so, directly require disclosure alongside other inherent risks.

## Conclusion

In this paper, I focused on non-transparent or, as I also called it, *black box AI* in medicine. More precisely, I discussed three of its characteristic risks and asked whether the physician needs to disclose them to patients, i.e. the risk of a cyber-attack, the risk of bias affecting a patient’s health care, and the risk of a mismatch. In each case, I argued that these risks require disclosure under certain circumstances or, otherwise, the physician either vitiates a patient’s informed consent or violates a more general obligation to warn him about potentially harmful consequences. I based my arguments on the assessment of the nature and likelihood of these risks together with replies against principled objections to making their disclosure a requirement. I also explained that these risks are exacerbated in the current COVID-19 pandemic and I thereby further emphasised their significance. Taken together, these claims aim to guide medical disclosure in clinical settings where AI plays an increasingly important role.

## Footnotes

1 I talk about ‘patient’ and ‘physician’ as in the context of medical treatment, but the same statements also apply to ‘participant’ and ‘investigator’ as in the context of clinical research.

2 See Berg & Appelbaum (2001: 55–57) who distinguish between the nature of a risk, narrowly understood as its objective harmful impact, and the magnitude of a risk, understood as the subjective impact of some harm on a person and his way of life. See also Herring, 2016*,* 174–175.

3 Berg adds that imminence is another factor for the evaluation of risk. Since imminence does not play a role in my following arguments, I omitted this aspect.

4 This view is also shared in many other jurisdictions, e.g. in Germany (Parzeller et al. 2007).

5 To clarify, by ‘bias’ I understand a prejudice that makes people depart from the truth. I do not refer to the term ‘bias’ as it applies to algorithms in a mathematical sense where it means a value added to a mathematical function.

6 Addressing the question of responsibility and whether there may be a responsibility gap would require a separate paper. So, I bracket this question here. See (Coeckelbergh 2020; Smith 2020; Tigard 2020).
